# A Preliminary Evaluation of the Pro-Chondrogenic Potential of 3D-Bioprinted Poly(ester Urea) Scaffolds

**DOI:** 10.3390/polym12071478

**Published:** 2020-06-30

**Authors:** Samuel R. Moxon, Miguel J.S. Ferreira, Patricia dos Santos, Bogdan Popa, Antonio Gloria, Ramaz Katsarava, David Tugushi, Armenio C. Serra, Nigel M. Hooper, Susan J. Kimber, Ana C. Fonseca, Marco A. N. Domingos

**Affiliations:** 1 Division of Neuroscience and Experimental Psychology, School of Biological Sciences, Faculty of Biology, Medicine and Health, The University of Manchester, Manchester Academic Health Science Centre, Manchester M13 9PL, UK; samuel.moxon@manchester.ac.uk (S.R.M.); nigel.hooper@manchester.ac.uk (N.M.H.); 2 Department of Mechanical, Aerospace and Civil Engineering, School of Engineering, Faculty of Science and Engineering, The University of Manchester, Manchester M13 9PL, UK; miguel.ferreira@manchester.ac.uk (M.J.S.F.); p96.bogdan@gmail.com (B.P.); 3 Centre for Mechanical Engineering, Materials and Processes, Department of Chemical Engineering, University of Coimbra, Rua Sílvio Lima-Pólo II, 3030-790 Coimbra, Portugal; p.santos0495@gmail.com (P.d.S.); armenio.serra@gmail.com (A.C.S.); 4 Institute of Polymers, Composites and Biomaterials—National Research Council of Italy, V.le J.F. Kennedy 54—Mostra d’Oltremare Pad. 20, 80125 Naples, Italy; angloria@unina.it; 5 Institute of Chemistry and Molecular Engineering, Agricultural University of Georgia, 240, David Aghmashenebeli Alley, Tbilisi 0159, Georgia; r.katsrave@agruni.edu.ge (R.K.); d.tugushi@agruni.edu.ge (D.T.); 6 Division of Cell Matrix Biology and Regenerative Medicine, School of Biological Sciences, Faculty of Biology, Medicine and Health, The University of Manchester, Manchester Academic Health Science Centre, Manchester M13 9PL, UK; sue.kimber@manchester.ac.uk; 7 The Henry Royce Institute, The University of Manchester, Alan Turing Building, Oxford Road, Manchester M13 9PL, UK

**Keywords:** 3D bioprinting, cartilage repair, tissue engineering, poly(ester urea), scaffold design

## Abstract

Degeneration of articular cartilage (AC) is a common healthcare issue that can result in significantly impaired function and mobility for affected patients. The avascular nature of the tissue strongly burdens its regenerative capacity contributing to the development of more serious conditions such as osteoarthritis. Recent advances in bioprinting have prompted the development of alternative tissue engineering therapies for the generation of AC. Particular interest has been dedicated to scaffold-based strategies where 3D substrates are used to guide cellular function and tissue ingrowth. Despite its extensive use in bioprinting, the application of polycaprolactone (PCL) in AC is, however, restricted by properties that inhibit pro-chondrogenic cell phenotypes. This study proposes the use of a new bioprintable poly(ester urea) (PEU) material as an alternative to PCL for the generation of an in vitro model of early chondrogenesis. The polymer was successfully printed into 3D constructs displaying adequate substrate stiffness and increased hydrophilicity compared to PCL. Human chondrocytes cultured on the scaffolds exhibited higher cell viability and improved chondrogenic phenotype with upregulation of genes associated with type II collagen and aggrecan synthesis. Bioprinted PEU scaffolds could, therefore, provide a potential platform for the fabrication of bespoke, pro-chondrogenic tissue engineering constructs.

## 1. Introduction

The number of patients at risk of suffering from cartilage-related diseases is predicted to expand significantly as a direct result of increased life expectancy and an aging global population. Recent studies show that osteoarthritis (OA), the most common form of degenerative joint disease, affects nearly 59 million people in the United States and European Union combined, with the numbers expected to double by 2020 [1]. Current clinical therapies, including microfracture, mosaicplasty and autologous chondrocyte implantation (ACI) have shown limited capacity for restoring normal phenotype and functionality to early-stage damaged cartilage [2,3,4]. Moreover, the efficiency of such techniques is still hindered by significant drawbacks, such as poor control over cell differentiation, limited repairable defect size, tissue availability and patient site morbidity [2,3]. In cases of end-stage OA, total joint replacement remains as the surgical standard allowing the reestablishment of some function and reduction in pain. However, in the long run, this solution can carry considerable practical and economic cost due to the high price and complexity of revision surgeries [5]. The low success rate of clinical therapies has, consequently, promoted a move towards new strategies for repairing damage to AC. One approach that has attracted significant interest is the application of tissue engineering (TE) to regenerate AC defects. More specifically, promoting tissue repair by designing a physical template (scaffold), either cellular or acellular, capable of integrating into the defect and promoting the adhesion and proliferation of autologous cells and their synthesis of new extracellular matrix (ECM), has been a major goal [6,7,8]. The scaffold plays a pivotal role by providing temporary mechanical support and regulating cellular activity towards guided tissue ingrowth [9]. To achieve this goal, scaffolds should be designed to mimic the complex multi-functional and multi-compositional organisation of the native tissue. From a manufacturing view-point, 3D bioprinting offers a platform for the precise control over the spatial distribution of cells and materials, allowing for the controlled uniformity or heterogeneity of the final structure [10]. This computer-controlled technique operates in a layer-by-layer fashion and can be combined with computer aided design (CAD) or medical imaging to produce patient-specific implants [11,12].

A wide range of natural and synthetic biomaterials have been used in combination with bioprinting to generate mono or multi-phasic scaffolds, capable of mimicking the structural composition of cartilage tissue [13,14,15,16]. Synthetic polymers, including polycaprolactone (PCL), poly(lactic acid) (PLA), poly(lactic-*co*-glycolic acid) (PLGA), poly(ethylene oxide terephthalate) (PEO), poly(butylene terephthalate) (PBT), polyurethane (PU), polyethylene oxide (PEO), and polyethylene glycol (PEG), have attracted great interest due to the tunability of their mechanical properties and degradation rates [17]. PCL, a linear aliphatic polyester approved by the Food and Drug Administration (FDA) for medical purposes, is the most reported scaffolding material in the literature [7,8,18,19,20,21]. Multiple research groups have taken advantage of the high thermal stability, processability, and blending ability of PCL to generate highly accurate 3D scaffolds for TE applications [19,22,23,24]. However, the use of PCL as a cell-compatible and cell-instructive material for AC tissue regeneration is still hindered by its slow degradation rate (i.e., from six to thirty six months as a function of molecular weight), release of acidic by-products and poor biomechanical performance [18]. Additionally, PCL scaffolds often have to be surface treated to increase hydrophilicity in order to maintain chondrocyte phenotype [25]. Biodegradable and bioabsorbable α-amino acid based poly(ester urea)s (AA-PEUs)have been proposed as a new class of polymers with enhanced bioactivity for TE [26,27]. Synthesis of AA-PEUs is relatively easy to achieve through poly-condensation of bis(α-amino acid)-alkylene diester monomers, which allow for a controlled hydrolytic degradation process [27]. The first attempt in the preparation of PEUs was reported by Huang and co-workers in 1979, but the low molecular weight of the material prevented its application in TE [28]. An alternative method for the synthesis of PEUs yielding high molecular weight and enhanced mechanical properties was later proposed by Katsarava with the introduction of an acid chloride of carbonic acid in the interfacial polycondensation reaction with a di-*p*-toluenesulfonic acid salt of a bis(α-amino acid)-alkylene diester [29]. This allows for the preparation of PEUs with improved physicochemical properties and potentially opens new opportunities in terms of processing technologies for tissue-engineered devices. Biodegradable PEU and PEU-based composites in the form of electrospun meshes and 3D printed scaffolds have recently been reported as adequate templates for TE, supporting the adhesion and proliferation of different cell lines (e.g., fibroblasts, osteoblasts, chondrocytes and epithelial cells) and the osteogenic differentiation of human mesenchymal stem cells (hMSCs) [30,31,32,33,34]. In this work, an extrusion-based bioprinter was used to demonstrate the potential of hydrophilic, leucine-based poly(ester urea) (PEU) materials as TE platforms for the generation of chondrogenic 3D constructs with precise control over the geometry and dimensions of extruded filaments. Through comparison with PCL scaffolds, the pro-chondrogenic potential of PEU scaffolds was explored, using analysis of cell viability, proliferation and expression of chondrogenic markers by TC28a2 human chondrocytes.

## 2. Materials and Methods

### 2.1. Materials

Poly(ε-caprolactone) (PCL, CAPA 6500, Mw = 50,000) in the form of 3 mm pellets was obtained from Perstorp Caprolactones (Cheshire, UK) and used as received. l-leucine (≥98%), *p*-toluenesulfonic acid monohydrate (*p*-TSA) were purchased from Sigma-Aldrich (St. Louis, MO, USA). Anhydrous sodium carbonate (Na_2_CO_3_) was purchased from Fisher Chemical (Loughborough, UK). Triphosgene was obtained from TCI Europe (Zwijndrecht, Belgium), and 1,6-hexanediol (99%) were purchased from Acros Organics (Geel, Belgium). Chloroform (CHCl_3_), and toluene were supplied by José Manuel Gomes dos Santos Lda (Odivelas, Portugal). CHCl_3_ was dried over calcium chloride and distilled prior use. Deuterated dimethylsulfoxide (DMSO-*d*_6_) was purchased from Eurisotop (Saint-Aubin, France). 

### 2.2. Synthesis of Poly(Ester Urea) (PEU) 

**Figure 1:**
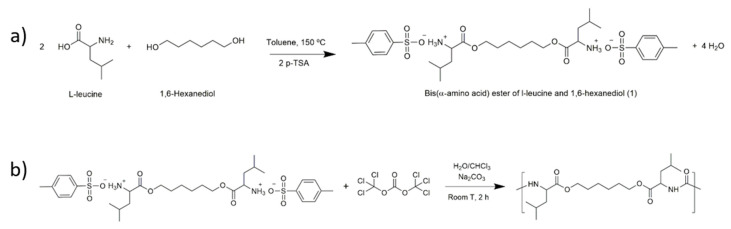
A representation of the chemical synthesis of PEU including (**a**) synthesis of bis(α-amino acid) ester from l-leucine and 1,6-hexanediol and (**b**) the reaction between bis(α-amino acid) ester from l-leucine and 1,6-hexanediol and triphosgene to yield the l-leucine-based PEU.

The synthesis of the PEU can be divided in two steps: (a) preparation of the bis(α-amino acid) ester and (b) reaction of the bis(α-amino acid) with triphosgene to yield the PEU. For the synthesis of the bis(α-amino acid) ester, a suspension of l-leucine (0.24 mol), 1,6-hexanediol (0.12 mol), and p-TSA (0.264 mol) in 300 mL of toluene was heated up to 150 °C with magnetic stirring, in a round bottom flask equipped with a Dean-Stark apparatus, and a condenser with a drying tube (Figure 1a). The suspension was heated to reflux until no more water was distilled. The excess of toluene was removed, and the resulting material was recrystallised twice using 300 mL distilled water, to yield 65 g of a white powder [35].

Bis(α-amino acid) ester of l-leucine and 1,6-hexanediol: yield: 80%; ^1^H-NMR (400 MHz,DMSO-*d*_6_): 0.90 (d, 12H) 1.34 (s, 4H) 1.61–1.71 (m, 8H) 2.30 (s, 6H) 3.97 (t, 2H) 4.15 (d, 4H) 7.13 (d, 4H) 7.49 (d, 4H) 8.31 (s, H).

For the synthesis of the PEU, the l-leucine based bis(α-amino acid) (0.008 mol), anhydrous sodium carbonate (1.8 g, 0.017 mol) and 80 mL of distilled water were placed in a 250 mL glass reactor, equipped with a mechanical stirrer. The mixture was stirred at 35 °C, for 30 min. The reactional system was cooled to 0 °C and anhydrous sodium carbonate (0.0085 mol) in 30 mL of distilled water was added to the reactor. After the reaction medium became transparent, triphosgene (0.003 mol) in 20 mL of dry CHCl_3_ was quickly added to the reactor. After 30 min, a solution of triphosgene (0.2 g, 0.7 mmol) in 6 mL of dry CHCl_3_ was added dropwise to the reactor. The reaction was allowed to proceed for 2 h (Figure 1b). After that time, the reaction mixture was placed in a separatory funnel. The organic phase was extensively washed with distilled water, and was then placed in a Teflon plate to allow the evaporation of CHCl_3_. Yield: 60%. *T*_g_ = 26 °C (see Figures S1 and S2 for the heat flow curve of PEU).

### 2.3. Scaffold Design and Fabrication

An extrusion-based 3D printing system (3D Discovery, regenHU, Switzerland) equipped with a screw-driven printing head and a 330 µm nozzle was selected for the fabrication of both PCL and PEU scaffolds. Adopting a methodology previously published by our group, rectangular prisms measuring 20 mm (length) × 20 mm (width) × 4 mm (height) were initially designed using BioCAD software (regenHU, Switzerland) and subsequently printed employing an optimised set of parameters (Table 1). The internal pore geometry (quadrangular) was defined by keeping a constant filament distance of 830 µm and alternating the deposition angle of adjacent layers between 0° and 90°. The obtained scaffolds were then cut into smaller specimens and used for further analyses.

### 2.4. Preparation of Films from the Scaffolds

The scaffolds were hot pressed in a laboratory hydraulic press (CARVER^®^), at 120 °C, and 0.1 metric tonne, for 15 min using cardboard moulds. The obtained films were then used in the contact angle measurements.

### 2.5. Characterisation of PEU and Scaffolds

#### 2.5.1. Identification of the Chemical Structure of the Poly(ester Urea) (PEU)

ATR-FTIR analysis of PEU was carried out with a Cary 630 FTIR spectrometer. Data collection was performed with 4 cm^−1^ spectral resolution and 64 accumulations in a 650 and 4000 cm^−1^ wavenumber range. ^1^H NMR spectra were obtained at 25 °C on a Bruker Avance 400 MHz Spectrometer using a 5 mm broadband NMR probe, in DMSO-*d*_6_. Tetramethylsilane was used as the internal standard.

#### 2.5.2. Morphological Analysis

The morphology of 3D printed PCL and PEU scaffolds was assessed using scanning electron microscopy (SEM, FEI Quanta 200) and micro-computed tomography (Micro-CT, SkySCan 1072, Aartselaar, Belgium). After gold-sputtering the polymeric scaffolds (EMITECH K550X Sputter Coater, France), top and cross section SEM micrographs were obtained under high vacuum conditions employing a voltage of 15 kV and a pressure of 3.2 × 10^−5^ Torr. ImageJ software (National Institute of Health, Bethesda, MD, USA) was subsequently used to evaluate the structural integrity of the scaffolds and consistency between theoretical (pre-defined in the BioCAD) and experimental values of RW and FG. Micro-CT analysis was performed using a rotational step of 0.9° over an angle of 180° in order to assess the porosity, surface area to volume ratio, and interconnectivity of the scaffolds. Skyscan software packages and Image J software were employed for the reconstruction of cross sections and 3D models.

#### 2.5.3. Mechanical Analysis

##### Nanoindentation Tests

Nanoindentation tests were carried out on the filaments of PCL and PEU scaffolds. All the measurements were performed in 1 mN to 5 mN load range using a Nanotest Platform (Micromaterials, UK) with a diamond pyramid-shaped Berkovich-type indenter tip. Trapezoidal load functions were used considering a load hold period of 20 s and a loading–unloading rate of 300 µN/s. Load–depth curves were recorded and hardness values were evaluated using the Oliver and Pharr method. Hardness (*H*) was determined according to Equation (1), where *P_max_* and *A_c_* are the applied peak load and the projected contact area at the specified load, respectively. The projected contact area *A_c_* was determined from the penetration depth, depending on the geometry of the tip.

(1) $$H=\frac{P_{max}}{A_{c}}$$

##### Compression Tests

PCL and PEU block-shaped scaffolds were characterised by a length (*l*) of 5.0 mm, a width (*w*) of 5.0 mm and a height (*h*_0_) of 6.0 mm. All the tests were performed at a rate of 1 mm/min up to a strain of 0.4 mm/mm, using an INSTRON 5566 testing system. The procedure used to evaluate the “apparent” stress and strain was described in a previous work [7,36]. The compressive modulus was evaluated as slope of the initial linear region of the stress–strain curve.

#### 2.5.4. Water Contact Angle Measurements

Contact angle testing of distilled water was conducted on both films and single filaments of PCL and PEU scaffolds using a DATAPHYSICS OCA 20 apparatus. Distilled water was dropped on films and single filaments in different sites and the contact angle was evaluated. In particular, CCD cameras recorded the process of the droplet dropping on the filament until disappearing gradually.

The baseline for a sessile drop contact angle was made at the liquid–solid interphase. Contact angles were evaluated using the ellipse method for the extraction of the drop profile, as described in the literature, and reported as mean value ± standard deviation [37,38].

#### 2.5.5. Cell Seeding

TC28a2 human chondrocytes were cultured to 70% confluence in supplemented DMEM (10% FBS, 2.5% l-glutamine) before being dissociated with trypsin and counted using an automated cell counter (Countess, Life Technologies, UK). Cells were seeded into PEU and PCL scaffolds (5 × 10^5^ cells/scaffold) and cultured for 21 days with media changed every other day (DMEM Glutamax™ supplemented with 10% foetal bovine serum, 37 °C, 5% CO_2_).

#### 2.5.6. Confocal Microscopy Imaging

Cell-loaded scaffolds were fixed with 4% paraformaldehyde in PBS at room temperature for 30 min, washed with PBS and incubated with 100 mM glycine in PBS for 1 h. Then, samples were permeabilised with 2.5% Triton X-100 in PBS for 45 min and blocked with 1% BSA for 30 min. F-actin was stained with rhodamine phalloidin (1:40 *v/v* in 1% BSA/PBS) (Invitrogen) for 1 h and cell nuclei were counterstained with 4′,6-diamidino-2-phenylindole (DAPI) (1 µg/mL in PBS) (Bio-Techne, UK) for 5 min. Samples were then imaged using a confocal laser scanning microscope (CLSM, Leica SP8, Leica Microsystems).

#### 2.5.7. Cell Viability

Cell viability within the scaffolds was analysed at 7, 14 and 21 days after seeding using a resazurin metabolism assay. Briefly, cell-loaded scaffolds were incubated in supplemented media containing 0.1 mg/mL resazurin for 3 h at 37 °C. Medium was then aspirated and transferred to a 96-well plate. Absorbance at 570 nm was measured for each sample in triplicate using a BioTek™ ELx800™ absorbance microplate reader (BioTek, UK).

#### 2.5.8. RT-PCR

RNA was extracted and purified from TC28a2 cells within PEU and PCL scaffolds using an RNeasy^®^ isolation kit after 7, 14 and 21 days in culture. The purity and concentration of isolated RNA was determined using a NanoDrop™ spectrophotometer (ThermoFisher, UK) before cDNA was generated using an iScript™ cDNA synthesis kit. A 20 μL qPCR reaction was prepared for each sample using 25 ng of cDNA and primers for COL1A1, aggrecan and COL2A1. PCR was then performed using a Quantstudio qPCR machine (Applied Biosystems, UK) and the relative expression for each gene was calculated using the 2^−^^ΔΔ^^CT^ method with GAPDH as reference housekeeping gene. 

### 2.6. Statistical Analysis

Statistical analyses were performed using one way ANOVA followed by Bonferroni post hoc tests, statistical differences were set at *p* < 0.05. 

## 3. Results and Discussion

### 3.1. Characterisation of the Chemical Structure of the l-Leucine-Based PEU

**Figure 2:**
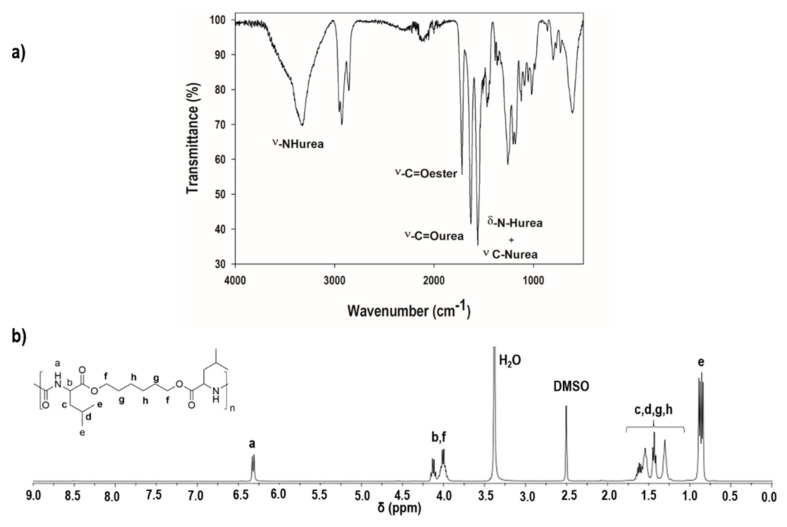
Spectroscopic data of the synthesised PEU demonstrating (**a**) the FTIR spectrum of PEU and (**b**) the ^1^H NMR spectrum of l-leucine-based PEU.

The chemical structure of the l-leucine-based PEU was studied by Fourier transform infrared (FTIR) and proton nuclear magnetic resonance (^1^H NMR) spectroscopies (Figure 2). In the FTIR spectrum, distinct bands corresponding to the ester and urea linkages were observed (Figure 2a). At 1726 cm^−1^, a band was detected ascribed to the stretching vibration of the –C=O_ester_. At ca. 3300 cm^−1^ and 1629 cm^−1^, it was also possible to isolate the bands corresponding to the stretching vibration of –NH_urea_ and –C=O_urea_, respectively. The band at ca. 1562 cm^−1^ was assigned to the bending vibration and stretching vibration of the –NH_urea_ and C-N_urea_. Further insights into the structure of the PEU were provided by ^1^H NMR analysis (Figure 2b). The resonance (a) at ca. 6.3 ppm corresponds to the protons of the –NH group of the urea linkage. The peaks observed between 3.9–4.25 ppm are ascribed to the protons–C*H*(C=O) (b) and –(C=O)OC*H*_2_– (f). The CH_2_ protons (c,g,h) and CH (d) protons resonate between 1.25–1.75 ppm. At ca. 0.8 ppm, the peak (e) can be identified, corresponding to the –C*H*_3_ protons. All the attributions are consistent with the anticipated chemical structure. Considering the results provided by the spectroscopic analysis, we concluded that the l-leucine-based PEU had been synthesised successfully. 

### 3.2. Morphological Analysis

**Figure 3:**
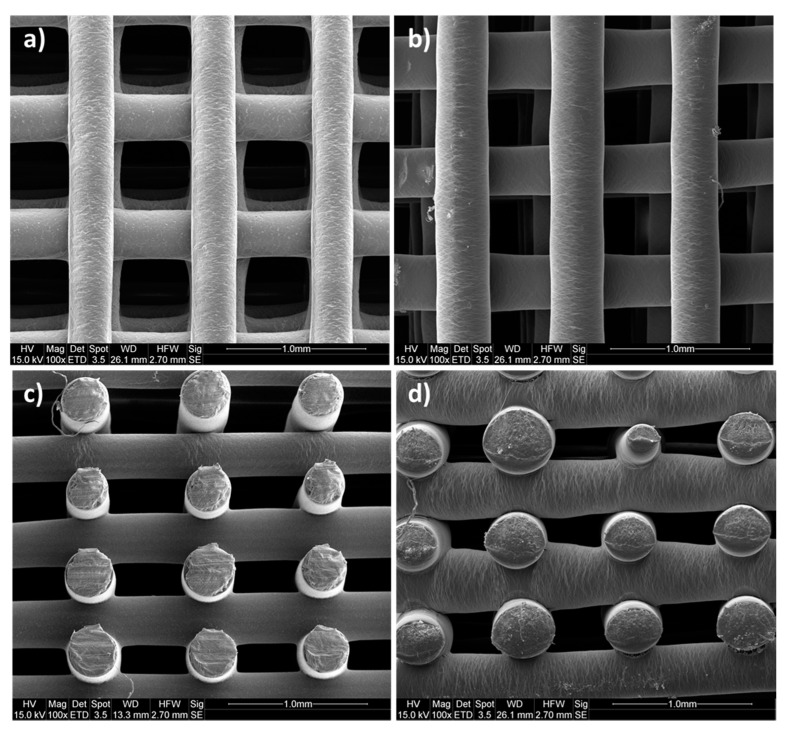
SEM micrographs of 3D printed scaffolds. (**a**) PCL scaffold top view; (**b**) PEU scaffold top view; (**c**) PCL scaffold cross-sectional view; (**d**) PEU scaffold cross-sectional view.

The interconnectivity and the distribution of pores together with pore geometry and size strongly influence cell distribution, proliferation and migration whilst shaping tissue ingrowth [7,36]. For this reason, the morphological features of PCL and PEU scaffolds were investigated by scanning electron microscopy (SEM) and micro computed tomography (Micro-CT). SEM micrographs demonstrated the successful printing of PCL and PEU scaffolds with well-defined square pore geometries and regular dimensions of approximately 500 µm × 500 µm (Figure 3). It is worth noting that the processing of PEU did not produce fibres as consistent in diameter as those produced for PCL. However, optimising process parameters still allowed for the extrusion of circular filaments with a fairly reproducible diameter (approx. 320 µm) and good adhesion between adjacent layers, thus ensuring the structural integrity of the constructs. 

Additionally, results from micro-CT analysis confirmed the generation of PCL and PEU scaffolds with a fully interconnected pore network and a repeatable microstructure with precise pore shape and size. Independently of the material, all constructs presented similar values of porosity (~63%) and surface area to volume ratio (13 mm^−1^). These data suggest a strong consistency was achieved between the values obtained from the experimental analyses and theoretical values defined during the scaffold fabrication (i.e., process and design parameters).

### 3.3. Mechanical Analysis

**Figure 4:**
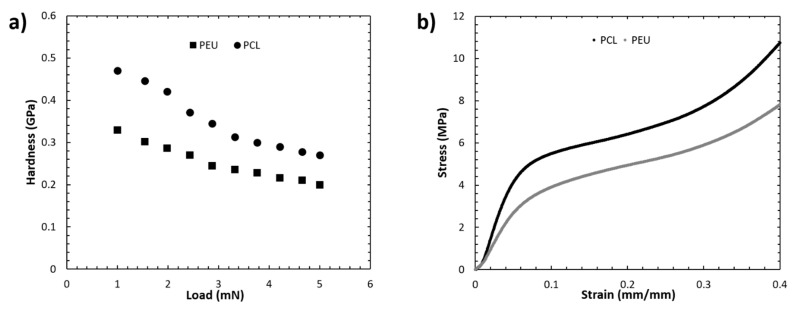
Mechanical data for 3D-bioprinted scaffolds of PEU and PCL (*n* = 5). (**a**) Nanoindentation data demonstrating the hardness of each scaffold as a function of the applied load, (**b**) compression data for each scaffold represented by stress–strain curves.

Nanoindentation measurements were conducted to facilitate analysis of the surface properties of the scaffold filaments. In comparison to microindentation techniques, nanoindentation allows for force displacement and application with much greater spatial resolution, leading to a better interpretation of localised surface hardness [39]. Data obtained from nanoindentation are influenced by the orientation and flexibility of polymer chains within the bulk structure [40]. Consequently, nanoindentation can be used to bridge the gap between atomic force microscopy and macroscale mechanical testing, allowing for the mapping of surface mechanical properties and assessment of microstructural features. Nanoindentation mapping of the bioprinted scaffolds revealed distinct mechanical properties between PCL and PEU filaments (Figure 4a).

In a load range of 1–5 mN, PCL filaments exhibited a higher hardness value (0.47 GPa–0.27 GPa vs. 0.33 GPa–0.20 GPa for PEU). Additionally, compression tests conducted on both scaffolds revealed stress–strain curves (Figure 4b) that were consistent with those previously reported for 3D printed PCL structures [7,36]. PCL and PEU scaffolds exhibited similar profiles of stress–strain curves; however, significant differences were observed for the bulk mechanical properties of the two scaffolds. More specifically, the values of compressive modulus (60.2 ± 1.4 MPa) and maximum stress (8.1 ± 0.2 MPa) achieved for PEU scaffolds were significantly lower than those obtained for PCL structures (79.8 ± 1.5 MPa and 10.9 ± 0.2 MPa, *p* < 0.001, *n* = 5). 

Combined, these data suggest that the bioprinted PEU scaffolds were significantly softer than the PCL scaffolds. This is of importance, as PCL is commonly applied in TE approaches to bone regeneration because the inherently stiff polymer provides mechanical cues to promote osteogenesis [41,42,43]. In contrast chondrogenic applications of PCL are often limited by the high matrix stiffness. Cartilage tissue is considerably softer than bone and, consequently, chondrogenesis has been reported to be enhanced on substrates with lower matrix stiffness leading to research groups often modifying PCL scaffolds to include a second, softer component [44,45,46,47]. The reduced hardness and compressive modulus of PEU scaffolds could, therefore, facilitate a better biological response from chondrocytes by providing a matrix stiffness that is more tailored to chondrogenic phenotype retention.

### 3.4. Water Contact Angle Measurements

**Figure 5:**
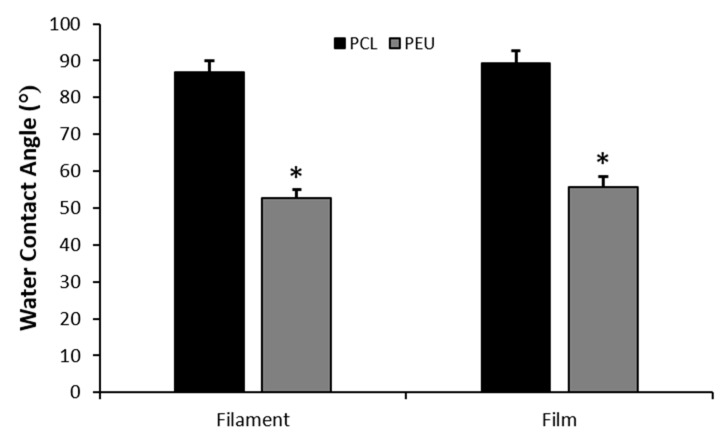
Average water contact angles for films and filaments of PCL and PEU scaffolds (* indicates *p* < 0.001, PEU vs. PCL; error bars represent standard error of mean, *n* = 5).

Wettability and hydrophilicity of both films and filaments of PCL and PEU scaffolds were investigated using water contact angle measurements (Figure 5). The technique involves introducing a liquid droplet to a surface and analysing the angle at which it interacts with the solid surface [48]. A contact angle <90° is indicative of a hydrophilic surface while contact angles of >90° represent hydrophobicity. 

Water contact angle values obtained for PEU filaments (52.7 ± 5.4°) were significantly lower than those obtained for PCL (86.9 ± 7.1°, *p* < 0.001). Additionally, the same trend was observed when comparing PCL and PEU films (89.3 ± 7.4° and 55.8 ± 6.0° for PCL and PEU respectively, *p* < 0.001). Consequently, PEU scaffolds appear to be significantly more hydrophilic than the PCL counterparts. This adds further strength to the application of bioprinted PEU scaffolds to cartilage TE, as multiple studies have demonstrated that hydrophilic substrates promote chondrogenesis by enhancing secretion and deposition of cartilage ECM by chondrocytes [49,50,51].

### 3.5. Biological Analysis

**Figure 6:**
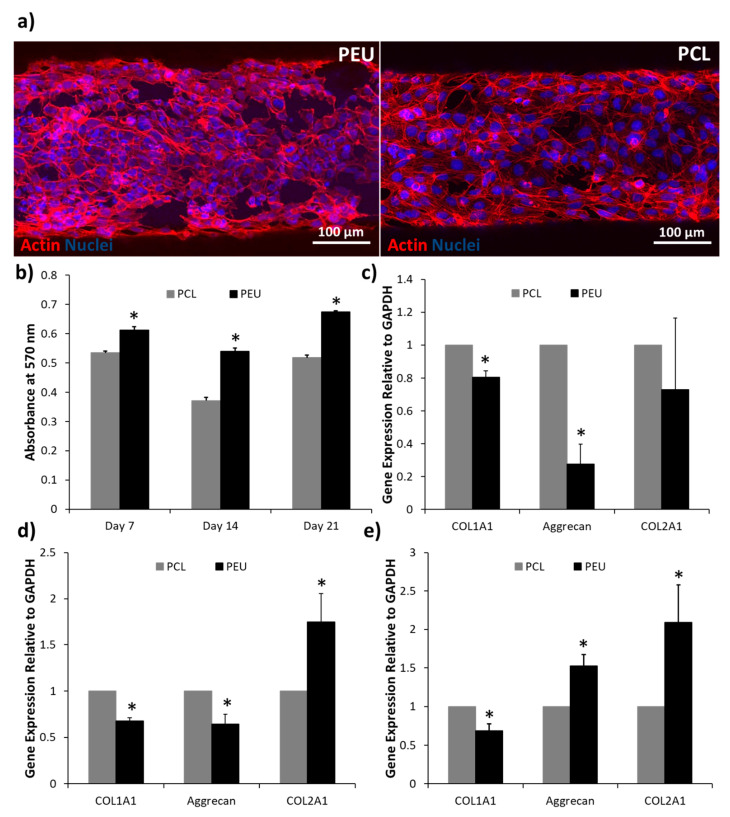
The biological response of TC28a2 human chondrocytes to PEU and PCL scaffolds. (**a**) Fluorescence confocal microscopy images of chondrocytes seeded onto PEU/PCL scaffolds and stained with phalloidin (actin) and DAPI (nuclei); (**b**) absorbance at 570 nm of chondrocyte culture media treated with 0.1 mg/mL resazurin after 7, 14 and 21 days on PEU and PCL scaffolds; (**c**–**e**) relative expression of COL1A1, aggrecan and COL2A1 by chondrocytes seeded on PEU and PCL scaffolds at day 7, 14 and 21, respectively (* indicates *p* < 0.05, PEU vs. PCL; error bars represent standard error of the mean, *n* = 3).

Single plain confocal microscopy images after phalloidin actin labelling indicated that both PEU and PCL scaffolds were supportive for TC28a2 human chondrocytes (Figure 6a). However, resazurin metabolism assays conducted at days 7, 14 and 21 revealed significant differences in cell viability between PEU and PCL constructs (Figure 6b). Chondrocytes cultured within PEU scaffolds absorbed significantly lighter at a wavelength of 570 nm. This is indicative of increased intracellular conversion of resazurin to resorufin via mitochondrial enzymes when cells were cultured within PEU scaffolds and, therefore, suggests the presence of a significantly higher number of viable cells [52]. In addition to this, chondrocytes cultured within PEU scaffolds exhibited differences in expression of chondrogenic markers when compared to cells culture in PCL scaffolds (Figure 6c–e). After 7 days of culture, chondrocytes exhibited significantly lower expression of genes associated with type I collagen and aggrecan synthesis, while levels of type II collagen were not significantly different (Figure 6c). At day 14 a similar trend was observed with the exception of type II collagen, which was expressed at significantly higher levels in the PEU scaffolds (Figure 6d). Finally, after 21 days in culture, a significantly higher expression of aggrecan and type II collagen was observed as well as a significantly lower expression of type I collagen (Figure 6e). At days 7 and 14 it can be argued that the differences between PEU and PCL are not distinct from a chondrogenic perspective. Type I collagen expression is significantly reduced in the PEU scaffolds within this timeframe and this is indicative of a more chondrogenic phenotype. However, at the same time points, aggrecan expression is significantly higher in PCL, which is also an indicator of a chondrogenic phenotype, while type II collagen expression does not show any differences until day 14. At day 21, however, a much more distinct, pro-chondrogenic effect is demonstrated in the PEU scaffolds, with lower type I collagen expression and higher expression of aggrecan and type II collagen. Combined, these data suggest a trend in gene expression that potentially demonstrates PEU better supports a more long-term chondrogenic phenotype in TC28a2 human chondrocytes than PCL [53,54]. This is likely due to chemical and mechanical differences between the two materials. Previous studies have demonstrated that hydrophilic surfaces enhance chondrogenic phenotypes by limiting protein adsorption and cell spreading, promoting a more spherical morphology [51,55,56]. Such a spherical phenotype is correlated with synthesis of a chondrogenic matrix [57,58,59]. Contact angle measurements revealed PEU to be significantly more hydrophilic than PCL and confocal microscopy images suggest that seeded cells exhibit reduced spreading on PEU scaffolds. Additionally, nanoindentation indicated that PEU was significantly softer than PCL, with hardness values of 0.33–0.20 GPa (versus 0.47–0.27 GPa) in the analysed load range (1–5 mN). This may also promote chondrogenesis, as prior studies have demonstrated that reduced matrix stiffness inhibits cell spreading and enhances expression of aggrecan and type II collagen [46,47].

## 4. Conclusions

This study highlights an initial evaluation of the pro-chondrogenic potential of bioprinted PEU scaffolds. Processing parameters for 3D printing were controlled such that high-resolution scaffolds of PEU with a porosity, surface area to volume ratio and fibre diameter close to that of an FDA-approved polymer, namely PCL, could be generated. Mechanical analyses revealed PEU scaffolds exhibited a significantly lower hardness and compressive modulus than PCL scaffolds. Additionally, water contact angle measurements demonstrated that the PEU scaffolds were significantly more hydrophilic than the PCL scaffolds. Combined, these properties stimulated the retention of a chondrogenic phenotype in TC28a2 human chondrocytes with an upregulation of COL2A1 and aggrecan and downregulation of COL1A1 when compared with cells seeded on PCL scaffolds after 21 days in culture. This is likely a direct result of the softer, more hydrophilic surface of PEU scaffolds promoting a spherical cell morphology, which is commonly reported to be critical in maintaining a chondrogenic phenotype. Moreover, previous studies have reported that PEU based scaffolds exhibit degradation properties that are favourable to applications for in vivo implantation [30]. However, further work will need to be conducted to investigate the effect of PCL and PEU molecular weight on biodegradation kinetics and cellular response. In conclusion, our results show that biocompatible AA-PEUs can be chemically synthesised to match the printability of PCL material whilst ensuring an enhanced chondrogenic phenotype in TC28a2 human chondrocytes.

## Figures and Tables

**Table 1:** Optimised set of process parameters used for the printing of PCL and PEU scaffolds. Parameters are denoted as deposition velocity (DV), slice thickness (ST), liquefier temperature (LT), extrusion pressure (EP) and screw rotation velocity (SRV).

Process Parameters					
	DV (mm/s)	ST (μm)	LT (°C)	EP (bar)	SRV (rpm)
**PCL**	20	280	90	5	11
**PEU**	22	280	125	5	8

## Supplementary Materials

The following are available online at https://www.mdpi.com/2073-4360/12/7/1478/s1, Figure S1: Heat flow curve (2nd heating cycle) of PEU; Figure S2: Heat flow curve (1st heating cycle) of PEU.
