# Supplementary material for: A Preliminary Evaluation of the Pro-Chondrogenic Potential of 3D-Bioprinted Poly(ester Urea) Scaffolds
Source: Polymers (Basel). 2020 Jun 30;12(7):1478. doi: 10.3390/polym12071478 (PMC7408263; doi:10.3390/polym12071478)
Supplement: Supplementary file 1 [file polymers-12-01478-s001.pdf]

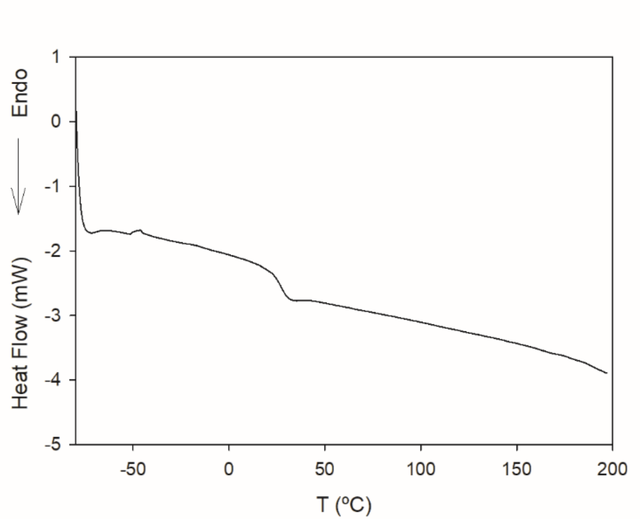

Figure S1: Heat flow curve (2nd heating cycle) of PEU.

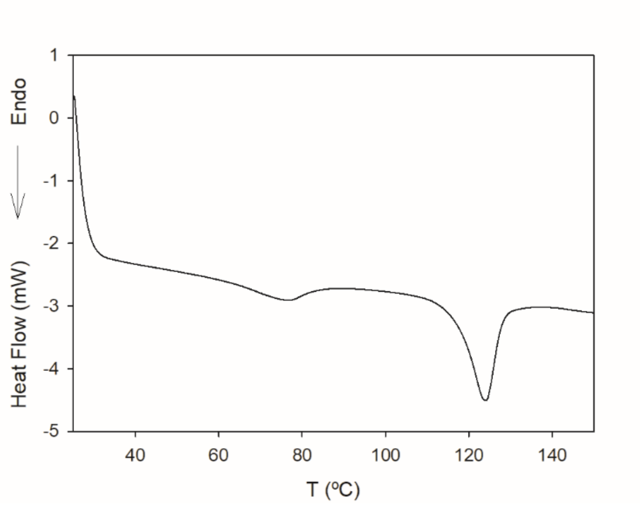

Figure S2: Heat flow curve (1st heating cycle) of PEU.
